# Supplementary material for: A Bayesian Perspective on Accumulation in the Magnitude System
Source: Sci Rep. 2017 Apr 4;7:630. doi: 10.1038/s41598-017-00680-0 (PMC5428809; doi:10.1038/s41598-017-00680-0)
Supplement: Supplementary file 1 — Supplementary Information Legends [file 41598_2017_680_MOESM1_ESM.pdf]

A Bayesian Perspective on Accumulation in the Magnitude System

Benoît Martin[1], Martin Wiener[2], Virginie van Wassenhove[1*]

[1]CEA, DRF/I2BM, NeuroSpin; INSERM, U992, Cognitive Neuroimaging Unit; Université Paris-Sud; Université Paris-Saclay, F-Gif/Yvette, France.[2]Department of Psychology, George Mason University, Fairfax, VA USA

SUPPLEMENTARY MATERIALS

VIDEOS

Linear video: In Experiment 1, a linear distribution of sensory evidence accumulation was tested during which dots appeared on the screen at an equal rate over time. This video displays three sequential trials in which participants have to judge the total duration, number and surface of the trial. Reponses were given on a scale with the positive and the negative extremes changing randomly across trials. The first trial display a total of 32 dots covering a surface of 595 mm^2 over 840 ms; the second trial display a total of 38 dots covering a surface of 476 mm^2 over 800 ms; the third trial display a total of 24 dots covering a surface of 500 mm^2 over 800 ms.

FastSlow video: In Experiment 2, a fast-slow distribution of sensory evidence accumulation was tested during which dots accumulated rapidly on the screen for the first 25% of the trial and more slowly for the remaining 75% of the trial. This video displays three sequential trials in which participants have to judge the total duration, number, and surface of the trial. Reponses were given on a scale with the positive and the negative extremes changing randomly across trials. The first trial display a total of 32 dots covering a surface of 490 mm^2 over 840 ms; the second trial display a total of 30 dots covering a surface of 613 mm^2 over 800 ms; the third trial display a total of 32 dots covering a surface of 613 mm^2 over 600 ms.

SlowFast video: In Experiment 2, a slow-fast distribution of sensory evidence accumulation during which dots accumulated slowly on the screen for 75% of the trial, and more rapidly thereafter. This video displays three sequential trials in which participants have to judge the total number, duration, and surface of the trial. Reponses were given on a scale with the positive and the negative extremes changing randomly across trials. The first trial display a total of 30 dots covering a surface of 613 mm^2 over 800 ms; the second trial display a total of 32 dots covering a surface of 367 mm^2 over 840 ms; the third trial display a total of 32 dots covering a surface of 539 mm^2 over 800 ms.
